# Dr. Andrei Yakovlev: Visionary, Leader, Iconoclast

**DOI:** 10.1186/1745-6150-3-10

**Published:** 2008-03-26

**Authors:** Yuriy Gusev, Leonid Hanin, Lev Klebanov, Alex Tsodikov, Nikolay M Yanev, Alexander Zorin

**Affiliations:** 1 Department of Surgery, University of Oklahoma Health Sciences Center,920 Stanton L. Young Blvd., WP 2140, Oklahoma City, Oklahoma 73104, USA; 2 Department of Mathematics, Idaho State University, Pocatello, ID 83209-8085, USA; 3 Department of Probability and Statistics, Charles University, Sokolovska 83, Praha-8, CZ 18675, Czech Republic; 4 Department of Biostatistics, School of Public Health, University of Michigan, 1420 Washington Heights, Ann Arbor, MI 48109-2029, USA; 5 Department of Probability and Statistics, Chair, Institute of Mathematics and Informatics, Bulgarian Academy of Sciences, 8 G.Bonchev, Sofia 1113, Bulgaria; 6 Department of Biostatistics and Computational Biology, University of Rochester Medical Center, 601 Elmwood Avenue, Box 630, Rochester NY 14642, USA

On February 27, 2008 the world's scientific community lost one of its most brilliant and innovative members – Dr. Andrei Yakovlev. He was 63, and at the zenith of his intellectual powers. With his uncanny ability to persuade and to ignite interest in his ideas, he challenged and stimulated everyone around him through his keen scientific vision and unparalleled intellectual depth and rigor. He collaborated with dozens of researchers – mathematicians, statisticians, biologists, epidemiologists, medical doctors and engineers. For many of them, these collaborations were a life changing experience. His unflinching belief in the power of mathematics and his tireless pursuit of new biomedical knowledge were irresistibly infectious and inspiring to two generations of his students and hundreds of his colleagues. For more than three decades he passionately advocated his vision for the future of Biology and Medicine as disciplines built upon a solid foundation of mathematical theory and statistical methodology. He has always enjoyed and cherished those wonderful "moments of truth" when predictions based on his models were confirmed by biological experiments or provided a perfect fit to known biomedical data, and he shared his joy with his co-workers, students and friends. He was a natural leader, caring mentor, and paternal figure for everyone privileged to work under his tutelage. He led by his wisdom, honesty, dignity and championship for scientific truth. He was a towering moral authority for many of his friends and coworkers, always ready to help, offer advice and even, for better or for worse, make critical decisions for them.

Andrei Yakovlev was born on November 30, 1944 in Leningrad (now St. Petersburg), Russia. The period of his adolescence and youth fell upon the era of the post-WWII revival of mathematics and natural sciences in the Soviet Union. The combination of good education, professional opportunity and relative freedom from the oppressive communist ideology attracted thousands of talented and daring young people to mathematics, theoretical and experimental physics, chemistry, biology and medicine. Andrei took full advantage of this unique historical chance and received an M.D. from the 1st Medical Institute in Leningrad (1967), a Ph.D. in cell biology from the Pavlov Institute of Physiology of the Academy of Sciences of the USSR (1973) and a Doctor of Science degree (which is the highest scientific degree in Russia) in Mathematics from Moscow State University (1981). With such a formidable background he was uniquely positioned to transcend the boundaries of the four different sciences, cultures and intellectual worlds of Mathematics, Statistics, Biology and Medicine.

Dr. Yakovlev made definitive and lasting contributions to a stunning number of fields: cell biology; cell population dynamics; branching stochastic processes; stochastic models in radiation biology; optimization of radiation cancer treatment; carcinogenic risk assessment; stochastic models of carcinogenesis, cancer growth, progression and detection; optimal schedules of cancer surveillance and screening; survival analysis; cancer epidemiology; statistical inference from microarray gene expression data; genetic regulatory networks; biostatistics; bioinformatics; and computational biology. His pioneering ideas were embodied in four research monographs and about 200 papers. He was the principal investigator on 12 major research grants, and since 1985 he gave more than 150 invited talks in the US and Europe alone. Yet he thought that his main tasks lay ahead of him as more and more groundbreaking ideas were taking shape in his mind, waiting to be tested, elaborated and implemented.

Dr. Yakovlev had an incredible intuition as to which biomedical problems are amenable to mathematical approaches and what is the right mathematical model or statistical test to employ. As one example, following A.N. Kolmogorov's groundbreaking works of the 1930–1940s on branching stochastic processes, Dr. Yakovlev made this approach a powerful tool in the study of cell proliferation kinetics and produced new results on stem cell differentiation that were theretofore too difficult even to contemplate. Furthermore, his work led to formulation of new concepts and discovery of new problems and directions of research in the mathematical theory of branching stochastic processes. As yet another example of the interdisciplinary impact of his ideas, his contributions to biomathematical modeling of carcinogenesis and tumor recurrence have sparked the development of new frequentist and Bayesian biostatistical methodology in cure models based on their hierarchical mixture origin, a field of biostatistics of general significance far beyond the initial focus on cancer.

Over the last seven years of his life Dr. Yakovlev developed a strong interest in microarray data analysis. In this very active area of research he designed a novel statistical methodology that allowed him to uncover unknown patterns of gene expression and interaction of great biological importance. In particular, this led him to the discovery of the major impact that the correlation structure of gene expression data has on the stability of multiple testing procedures – a fact that has never been noticed before.

His scientific legacy does not consist only of ideas, methods and results. Most importantly, he left behind scientific principles and research paradigms that will guide his co-workers and students for decades to come. He forged them through incisive analysis, trial and error, and excruciating, sometimes heated and fierce, debates with his colleagues and disciples. He has never articulated them explicitly, perhaps leaving this endeavor for a future time, but followed them religiously in his own work.

Dr. Yakovlev has held numerous leading positions throughout his distinguished scientific career. In 1978 he founded the department of Biomathematics in the Central Research Institute of Roentgenology and Radiology (Leningrad, Russia) and headed it until 1988. From 1988–1992 he chaired the Department of Applied Mathematics of Leningrad (later St. Petersburg) State Polytechnical University. In 1992–96 he held visiting professorships at several universities and research institutions in the US, France, Germany and Australia. In 1996 he was appointed to the position of Director of Biostatistics at the Huntsman Cancer Institute of the University of Utah and settled in Salt Lake City. His last academic post was at the University of Rochester Medical Center, which recruited him as a founding chairman of the Department of Biostatistics and Computational Biology, a labor of love for the last six years of his life. As a result of his tireless effort, the department has seen a three-fold expansion and a six-fold increase in external research funding, which placed it among the world's top departments in the field.

Dr. Yakovlev's academic life spanned several countries (Russia, Germany, France and the US) leaving in his wake numerous admirers, disciples, followers and co-workers as well as those apprehensive of his fierce pursuit of scientific truth, relentless questioning of conventional wisdom, zero tolerance for junk science, and little patience for scientific orthodoxy. Many of his ideas, especially in the fields of biostatistics, cancer epidemiology, cell biology and microarray data analysis, appeared decades ahead of their time. Due to their revolutionary nature, mathematical sophistication and broad interdisciplinary scope, they were found inaccessible by some of his peers. With the system of anonymous peer refereeing currently in place, which Dr. Yakovlev considered fundamentally broken, some of his best and most innovative papers and grant proposals were undervalued by narrowly specialized reviewers. That is why Dr. Yakovlev embraced with great enthusiasm the new framework of open peer reviewed publication pioneered by BMC-Biology Direct and became the founding editor of its Mathematical Biology section.

It is comforting to us that his exceptional research accomplishments were recognized by many prominent national and international organizations. He was a recipient of the Alexander von Humboldt Award (1994) and a John Simon Guggenheim Fellowship (1999) and was named elected member of six academies and societies. In recognition of his important contributions to statistics, he was named Honorary Fellow of the Institute of Mathematical Statistics (1998), Fellow of the American Statistical Association (2000) and Elected Member of the International Statistical Institute (2002). Among many honors bestowed on Dr. Yakovlev are Honorary Doctor of Science of Idaho State University (2002), Distinguished Scholarly and Creative Research Award of the University of Utah (2002) and the title of Distinguished Lecturer of Creighton University (2006).

**Figure 1:**
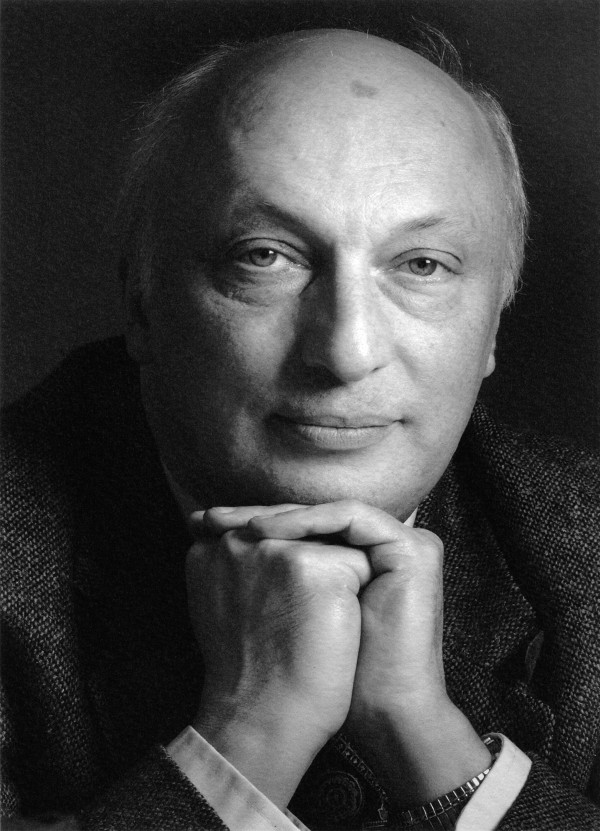
A recent photograph of Dr. Yakovlev.

We believe that with the passage of time the broad scientific community will come to realize that in Dr. Yakovlev (figure 1) we have lost one of the greatest scientific visionaries of our time. It is heart wrenching that he did not live long enough to witness this recognition.

He is survived by his wife Nina and their 11 year old son Yuri.

